# Effects of Banana (*Musa* spp.) Bract Flour on Rats Fed High-Calorie Diet

**DOI:** 10.17113/ftb.61.02.23.7762

**Published:** 2023-06

**Authors:** Isabela Rezende Ferreira, Valfredo de Almeida Santos Junior, Édina Caroline Ferreira Almeida, Felipe Francisco Bittencourt Junior, Ariany Carvalho dos Santos, Virginia Demarchi Kappel Trichez, Elisvania Freitas dos Santos, Mariana Manfroi Fuzinatto, Priscila Neder Morato

**Affiliations:** 1 Department of Food Science and Technology, Federal University of Grande Dourados, João Rosa Góes Street, Vila Progresso, 79825-070 Dourados, MS, Brazil; 2 Department of Food and Nutrition, State University of Campinas, University City Zeferino Vaz, Barão Geraldo, 13083-970 Campinas, SP, Brazil; 3 Department of Biological and Health Sciences, University Center of Grande Dourados, Balbina de Matos Street, Jardim Tropical, 79824-900 Dourados, MS, Brazil; 4 Department of Health Sciences, Federal University of Grande Dourados, João Rosa Góes Street, Vila Progresso, 79825-070 Dourados, MS, Brazil; 5 Department of Pharmaceutical Sciences, Food and Nutrition, Federal University of Mato Grosso do Sul, University City, Costa e Silva Street, 79070-900 Campo Grande, MS, Brazil; 6 Department of Engineering, State University of Mato Grosso do Sul, Emílio Mascoli Street, Jardim Vale Encantado, 79950-000 Naviraí, MS, Brazil

**Keywords:** banana plant waste, antioxidant activity, high-calorie diet, hepatoprotective effect

## Abstract

**Research background:**

The extensive cultivation of bananas (*Musa* sp.) is related to producing tons of residues, such as leaves, pseudostems and bracts (inflorescences). The banana bract is a commercially interesting residue due to its dietary fibre content and high antioxidant potential. With this in mind, this study evaluates the effects of administering banana bract flour in animal models fed a cafeteria diet.

**Experimental approach:**

Thirty-two male rats were divided into 4 groups: (*i*) control diet, (*ii*) control diet with 10 % banana bract flour, (*iii*) hypercaloric diet, and (*iv*) hypercaloric diet with 10 % bract banana flour. The study was conducted for 12 weeks and included analysis of phenolic compounds, assessment of the antioxidant effect of banana bract flour, determination of serum biochemical parameters (glucose, total cholesterol, triglycerides, aspartate aminotransferase (AST), alanine transaminase (ALT), amylase, albumin, uric acid, creatine, total protein, and oral glucose), determination of faecal fat content, and histomorphological analysis of the liver, pancreas and adipose tissue. In addition, molecular parameters such as IL6, total and phosphorylated JNK, total and phosphorylated IKKβ, TNFα, TLR4 and HSP70 were determined.

**Results and conclusions:**

The banana bract flour showed a high content of phenolic compounds and an antioxidant effect. The *in vivo* results suggest that the supplementation of a hypercaloric diet with banana bract flour prevented pathological damage by reducing total cholesterol and glucose amounts, which may imply a hepatoprotective effect of this supplement. Thus, using banana bract flour as a supplement can increase the consumption of fibre, antioxidants and bioactive compounds.

**Novelty and scientific contribution:**

The development of flour from banana waste and its inclusion in the diet can prevent and/or help treat obesity. In addition, the use of banana bracts can help protect the environment, as they are considered a source of waste by the food industry.

## INTRODUCTION

Low energy expenditure combined with high energy intake may result in hypertrophy and hyperplasia of adipocytes, which can lead to tissue hypoxia, increased production of free radicals (*1*), and consequently, increased secretion of pro-inflammatory adipocytokines by the adipose tissue (*2*). In this scenario, the biological potential of some by-products of the food sector has attracted the attention of academic and industrial communities due to their potential functionality. For example, in banana bracts, the antioxidant action is mostly linked to the presence of phenolic compounds generated by the plant's secondary metabolism (*3*). In addition to providing antioxidant and bioactive substances, food by-products can be used as a source of fibre, an important nutrient related to satiety and the maintenance or prevention of lean mass gain (*4*).

**Fig. S1:**
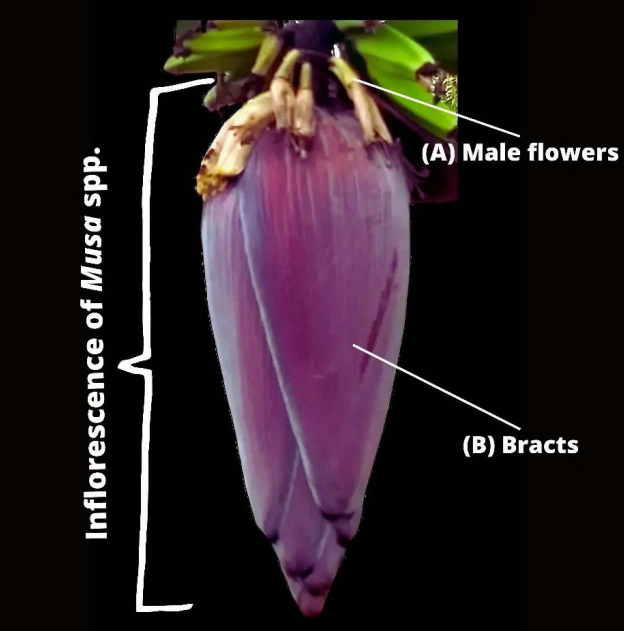
Inflorescence of *Musa* spp.: male flowers (A) and bracts (B)

Banana (*Musa* spp.) is an important food crop widely produced and consumed because of its pleasant taste, high nutritional value and health benefits (*5*). Furthermore, the banana inflorescence (Fig. S1), also known as banana "blossom", "flower" or "heart", is a structure that includes male flowers with their specific reddish-purple bracts (*5*, *6*). However, in extensive cultivation, the banana bract is generally discarded right after its opening for better fruit development. Because of this, banana inflorescence is a major waste in banana crops (*7*).

The banana inflorescence is commonly eaten worldwide, complementing a variety of dishes in Asian countries, in fried versions, seasoned with curry, or in boiled form (*8*, *9*). However, in Brazil, the consumption of banana bract is still discreet, reports associate it more with rural areas in the form of nutritious snacks, pies or added cooked in salads to increase the yield of meals (*10*).

Antidiabetic and antilipidemic properties of banana inflorescence extract have been reported previously (*11*, *12*). In addition, banana bract has high amounts of fibre and antioxidant effects, representing a viable and cost-effective source of bioactive compounds (*8*, *13*). Therefore, bearing this in mind, the banana bract may represent a nutritious and functional food source, making its consumption highly recommended and viable, especially due to the ease of its addition to meals as dehydrated flour (*10*).

Due to the potentially positive effects of banana bract consumption against metabolic diseases, this study evaluates the potential effects of banana bract flour on obesity prevention and as a possible hepatoprotective agent in animal models fed a hypercaloric diet (modelled as cafeteria diet).

## MATERIALS AND METHODS

The acquisition and processing of banana (*Musa* spp.) bracts to obtain the flour (Dourados, Brazil), as well as the analysis of the centesimal composition of the final product, is described in our previous study (*14*).

### Phenolic content and antioxidant activity analyses

The sample extract was prepared with an extraction solution containing *φ*(ethanol)=70 % (Sigma-Aldrich, Merck, St. Louis, MO, USA) (*7*).

The total phenolic compounds of the banana bract flour were determined using the Folin-Ciocalteu method, with the modifications proposed by Asami *et al*. (*15*), adopting repeated measurements of the samples (triplicates). To determine the phenolic compounds, aliquots with 200 μL of extract, 60 μL of Folin-Ciocalteu reagent (Sigma-Aldrich, Merck), and 2 mL of sodium carbonate solution (*φ=*7 % (Sigma-Aldrich, Merck) were used. The absorbance of the reagent mixture was measured at *λ*=720 nm using a spectrophotometer with a UV-Visible working range (model UV-1600; PRO-TOOLS, Shanghai, PR China). Quantification was performed using a calibration curve prepared with a standard solution of *γ*(gallic acid)=72–200 μg/mL (São Paulo, Brazil), with results expressed in mg of gallic acid equivalent (GAE) per g of dry matter.

The total antioxidant activity of the flour was determined by the DPPH (1,1-diphenyl-2-picrylhydrazyl) radical (Sigma, Suzano, Brazil) method, as proposed by Brand-Williams *et al.* (*16*), with modifications. Briefly, 0.1 mL of sample extracts at different concentrations were diluted in 70 % ethanol (Sigma-Aldrich, Merck). Then, the volume was completed to 5 mL with methanol, homogenised, and kept in the dark for 30 min. Finally, absorbance was measured at *λ*=515 nm using a spectrophotometer with a UV-visible working range (model UV-1600; PRO-TOOLS).

The DPPH radical scavenging activity of the sample was expressed as Trolox equivalent antioxidant capacity (TEAC) in μmol of Trolox per g of sample.

### Animals

The Ethics Committee of the Federal University of Grande Dourados (UFGD), Dourados, Brazil, approved the experimental procedures for research with animal models under protocol no 38/2017. All procedures followed the ethical principles of animal experimentation established by the Brazilian School of Laboratory Experimentation (SBCAL/COBEA) (*17*).

Thirty-two male Wistar rats (*Rattus norvegicus*), 5-6 weeks old, were used in this study. The animals from the central animal facility (vivarium) of the UFGD were housed in the animal facility of the Faculty of Health Sciences, UFGD, in polypropylene cages for rodents (CHOCMASTER®, Paraná, Brazil). Four or five male rats were allocated per cage and placed under controlled environmental conditions: (22±1) °C temperature, 50-60 % humidity, and 12-hour light/dark cycles. Water and diet were provided *ad libitum*.

### Diet and interventions

Male animals (Dourados, MS, Brazil) were weighed (Japan SLC, Inc., Shizuoka, Japan) on the first day of the experiment and randomised into four experimental groups (*N*=8). Four distinct diets were offered to the four distinct groups formed: (*i*) control diet (C) (commercial feed, Labina, Purina, São Paulo, Brazil), (*ii*) control diet with bract flour (CB), (*iii*) high-calorie cafeteria-type diet (CAF), and (*iv*) high-calorie cafeteria-type diet with bract flour (CAFB). Water and diet were offered *ad libitum* for twelve weeks. The CAF and CAFB diet groups also had soft drinks (AMBEV, Sete Lagoas, Brazil) offered *ad libitum* (*18*). In addition, bract flour was added at a rate of 10 %, as established by Liyanage *et al*. (*19*) and Bhaskar *et al*. (*20*).

The control diet (C) was composed of 100 % commercial feed (100 g/100 g). The CB diet was composed of commercial feed (100 g/100 g) and banana bract flour (10 g/100 g). The CAF diet consisted of commercial chow (37.5 g/100 g), roasted peanuts (25 g/100 g; Yoki, São Bernardo do Campo, Brazil), chocolate powder (25 g/100 g; Dr Oetker, Curitiba, Brazil) and wafer biscuits (12.5 g/100 g; Bauducco, Guarulhos, Brazil). Finally, the CAFB diet consisted of commercial chow (37.5 g/100 g), roasted peanuts (25 g/100 g), chocolate powder (25 g/100 g), wafer biscuits (12.5 g/100 g), and banana bract flour (10 g/100 g). The CAF diet was adapted from Estadella *et al.* (*21*). The diet ingredients were manually mixed and moulded into small cubes, then placed in an oven at 70 °C until dry. The diets were analysed for centesimal composition with a methodology similar to bract flour analysis (*14*). The total energy value was calculated with the following values: lipids 37.78 kJ/g, proteins 17.86 kJ/g and carbohydrates 15.98 kJ/g (*22*). Mean results were expressed as percentage value±standard deviation of the mean.

Considering the suppliers, the dietary fibre content of the diets was theoretically calculated (*23*). The commercial feed used had approx. 7 % and roasted peanuts 8 % of total dietary fibre per 100 g product. Therefore, except for bract flour added in the intervention groups, commercial feed and roasted peanuts were considered the major ingredients in the theoretical calculation of dietary fibre content; the other ingredients were not considered. In addition, the animal mass, food consumption and water consumption were checked daily.

### Intraperitoneal glucose tolerance test

An intraperitoneal glucose tolerance test (ipGTT) was performed on fasted rats (12 h) after 8 weeks of treatment. First, blood glucose concentrations were measured using a glucometer (FreeStyle Lite®; Abbott Diabetes Care, Abbott Laboratories, Brazil). For the test, blood glucose concentrations were recorded at time 0 as the baseline value. Afterwards, rats received intraperitoneally a solution containing 50 % d-glucose (2 g per kg body mass; Neon, Suzano, Brazil). Blood samples were collected from the tail vein at 15, 30, 60 and 120 min. Finally, the area under the curve was calculated (*24*).

### Analysis of fat in the stool

The stool of all different groups was collected at week 11, for three consecutive days, for lipid content analysis using the Soxhlet method (ST 243 Soxtec™; FOSS, Hillerød, Denmark). The weighted samples of 5 g were placed into a cellulose thimble and dried to eliminate any excess moisture. Hexane (Sigma-Aldrich, Merck) was used as an extracting solvent. The extraction process was continued for about 4 h at 70 °C. After extraction, the solvent was evaporated, dried, and weighed gravimetrically (*25*).

### Naso-anal length

The naso-anal length (NAL) of animals was checked at the end of the experiment with an anthropometric tape (Sanny®, São Bernardo do Campo, Brazil).

### Blood and tissue collection for biochemical analysis

At the end of the experiments, all animals were forced to fast for 8 h (*26*) and then they were euthanised by inhalation anaesthesia with isoflurane (Sigma-Aldrich, Merck), followed by decapitation. Blood samples were collected in polyethene tubes (FirstLab®, São José dos Pinhais, Brazil) and polyethene tubes with EDTA anticoagulant (FirstLab®).

Liver, heart, epididymal and retroperitoneal fat were removed, weighed on an analytical scale, and macroscopically analysed. The mass fraction of each organ was calculated according to the following equation:

*w*(organ)=(*m*(organ)/*m*(body))·100 /1/

### Blood parameters

The biochemical parameters were analysed by a Cobas C111 spectrophotometer (Roche Diagnostics® GmbH, Mannheim, Germany) using commercial kits (Roche Diagnostics®, Basel, Switzerland). The Cobas C111 analyser uses a single-point calibration, creating a calibration curve based on one standard. The machine was calibrated with a calibrator for an automated system, and the instrument software produced the calibration curve. The biochemical parameters were evaluated at *λ*/nm: total cholesterol 583–659, glucose 340–409, triglyceride 512–659, amylase 660, uric acid 552–659 creatine 512–583, alanine aminotransferase (ALT) 340–378, aspartate aminotransferase (AST) 340–378, albumin 583–512 and total protein 512.

The haematological analyses were performed using the KX-21 automation unit (Sysmex, Kobe, Japan) and reviewed in a slide using differential counting (leukocytes) and observing cell morphology/staining. The following haematological parameters were measured: leukocytes, haematocrits, platelets and lymphocytes.

### Histopathological analysis

Liver, pancreas, and adipose tissue fragments were fixed in 10 % buffered formalin (Sigma-Aldrich, Merck). After fixation, they were cleaved, dehydrated in ethanol (Sigma-Aldrich, Merck), diaphanized in xylene (Sigma-Aldrich, Merck), embedded in paraffin (Santa Cruz, São Paulo, Brazil), and sectioned using a section cutter (Leica, Davisburg, MI, USA) at a thickness of 4 μm. Haematoxylin and eosin (Sigma-Aldrich, Merck) staining was performed for morphological observation using a system incorporated in the Carl Zeiss microscope (ZEISS®, Jena, Germany) (*27*).

### Western blot

For the determination of molecular parameters using the Western blot method, the gastrocnemius muscle was removed after euthanasia. The protein content present in the tissue homogenate after polytroning (Polytron® model 713T; Fisatom Scientific Equipment, São Paulo, Brazil) was determined by the Lowry method (*28*). The samples were prepared by homogenising ~100 mg of frozen tissue in Triton buffer (100 mM Tris, pH=7.4, 1 % Triton X-100) containing 100 mM tetrasodium diphosphate, 100 mM NaF, 10 mM EDTA, 10 mM Na_3_VO_4_, 2 mM phenylmethanesulfonyl fluoride (PMSF) and 0.1 mg/mL aprotinin (all reagents from Sigma-Aldrich, Merck). The total protein content of the muscle samples was determined. For immunoblotting, tissue homogenates were subjected to sodium dodecyl-sulfate polyacrylamide gel electrophoresis (SDS-PAGE) and transferred to nitrocellulose membrane by trans-blot semi-dry electrophoretic transfer cell (Bio-Rad®, Hercules, CA, USA). The membranes were incubated with the antibodies to determine IL6, total and phosphorylated c-Jun NH2-terminal kinase (JNK), total and phosphorylated kB kinase-B (IKKβ), TNFα, TLR4 and HSP70 proteins. The loading control with β-actin was used to assess the amounts of the immunological proteins analysed in the muscle. The bands were visualised by chemiluminescence imaging systems using the UVITEC equipment photo documenter (Uvitec Alliance 4.7®, Cambridge, UK) and quantified using ImageJ® (v. 1.44 for Windows) software (*29*).

### Statistical analysis

Results were expressed as mean value±standard deviation of the mean (S.D.) or mean value±standard error of the mean (SEM). All parameters of the male animals, including diet composition, were analysed for statistical significance (ANOVA and Tukey’s *post-hoc* tests p<0.05). The *t*-test was used to compare the C and CB groups and the CAF and CAFB groups. Statistical analyses were performed using the SPSS®, v. 11.0 (*30*). The compiled and analysed data were presented as tables and graphs using GraphPad Prism® v. 5 for Windows software (*31*).

## RESULTS AND DISCUSSION

### Banana bract flour phenolic content and antioxidant activity

A mass of 4.2 kg of flour was obtained from processing 63.3 kg of banana bract, a yield rate of 6.64 % (data not shown). The total phenolic mass fraction, expressed as gallic acid, found in banana bract flour was 556.51 mg/100 g (data not shown), a high value compared with the result of 106.24 mg/L obtained by Rodrigues *et al.* (*32*).

The antioxidant activity value for banana bract flour expressed as Trolox equivalents was (27.79±2.7) μmol/g (data not shown), which indicated a free radical scavenging potential of DPPH. Similar results were found by Begum and Deka (*8*) when analysing banana bract methanolic extract (80 %) also by DPPH, whose values for antioxidant activity expressed as Trolox equivalents on fresh mass basis were 24.53 and 27.96 μmol/g, for the inner and outer bract, respectively.

### Experimental diet centesimal composition and energy value determination

The experimental diet composition and energy value are shown in Table 1. The control diet showed the highest ash mass fraction ((7.14±0.04) g/100 g), and protein mass fraction similar to the CB diet. On the other hand, CAF and CAFB diets showed the highest values for lipid mass fraction without statistical differences. As expected, the CAF diet had the highest carbohydrate mass fraction and energy value (EV).

**Table 1:** Experimental diet centesimal composition and energy value

**Composition**	***w*/(g/100 g)**			
C	CB	CAF	CAFB
**Moisture**	(8.23±0.06)^cB^	(18.2±0.1)^aA^	(9.3±0.3)^b^	(9.0±0.1)^b^
**Ash**	(7.14±0.04)^a^	(6.92±0.05)^b^	(3.13±0.02)^dB^	(3.68±0.01)^cA^
**Protein**	(20.964±0.6)^a^	(18.8±0.9)^a^	(11.5±1.2)^b^	(12.4±0.9)^b^
**Lipid**	(4.6±0.2)^b^	(4.6±0.2)^b^	(15.1±0.2)^a^	(15.1±0.6)^a^
**Carbohydrate**	(52.1±0.5)^cA^	(38.9±0.1)^dB^	(56.5±0.9)^aA^	(49.6±0.2)^bB^
**Fibre**	7.00	12.61	4.62	10.23
**EV/kJ**	(1379.88±2.29)^cA^	(1129.68±4.18)^dB^	(1681.97±12.55)^aA^	(1585.74±8.36)^bB^

C=group with a standard diet (control), CB=group with standard (control) diet supplemented with 10 % bract flour, CAF=group with cafeteria diet, CAFB=group with cafeteria diet supplemented with 10 % bract flour, EV=theoretical energy value was calculated for 100 g of diet. Values were presented as mean±standard error of the mean (SEM), *N*=3. Different lowercase letters in the same row indicate significant differences at p<0.05 among groups (ANOVA and Tukey’s test). Different capital letters in the same row indicate significant differences at p<0.05 between groups (C+CB) and (CAF+CAFB) (*t*-test). The theoretical calculation was performed for carbohydrates and fibre

The EV for high-calorie diets ranged between the values found by Nascimento *et al*. (*33*) (15.27 kJ/g) and Navarro *et al*. (*34*) (22.17 kJ/g). An increase in total fibre mass fraction, and a reduction in both carbohydrate and CV was noted with the addition of banana bract flour when comparing control and CB diets and CAF and CAFB diets.

### Effects on physiological parameters of animals

**Fig. 1:**
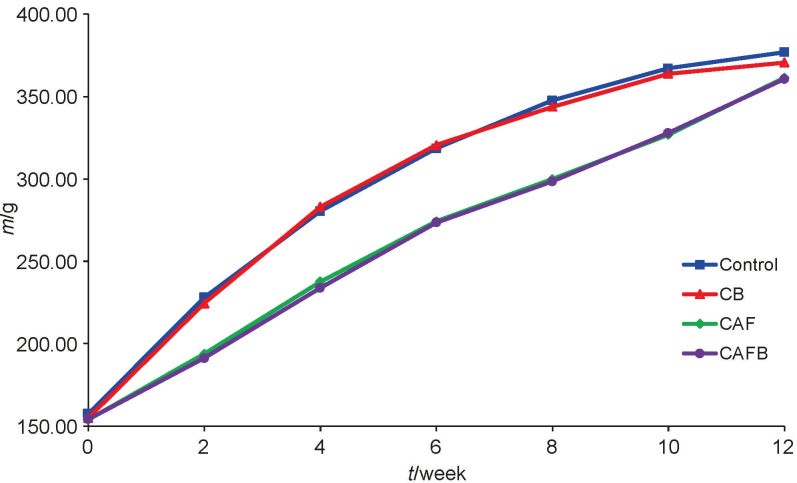
Weekly average body mass of the control and experimental groups. C=group with a standard diet, CB=group with standard diet supplemented with 10 % bract flour, CAF=group with cafeteria diet, CAFB=group with cafeteria diet supplemented with 10 % bract flour

All animals had ponderal growth (Fig. 1) comparable to the control group. However, the final mass and naso-anal length (NAL) of the animals did not change (at p<0.05) compared to the group that consumed only a commercial diet (Table 2).

**Table 2:** Growth parameters, feed and water intake, mass fraction of lipids in stool, and organ and fat relative mass in experimental groups

**Parameter**	C	CB	CAF	CAFB
***m*(initial)/g**	157.50±6.2	155.1±5.8	154.0±8.9	154.2±7.2
***m*(final)/g**	376±8	370±5	361±4	360±6
***l*(NAL)/cm**	22.9±0.3	22.7±0.3	22.2±0.2	22.3±0.2
Feed intake/(g/24 h)*	(21.2±0.8)^ab^	(23.90±0.02)^a^	(14.2±0.9)^cB^	(20.2±0.6)^bA^
Energy value/(kJ/24 h)**	(292.88±0.41)^bA^	(270.70±12.55)^cB^	(238.06±20.92)^dB^	(319.65±25.10)^aA^
**Water intake/(mL/24 h)**	(108.3±2.8)^cB^	(150.0±5.6)^aA^	(80.6±2.8)^dB^	(122.8±5.6)^bA^
***m*(stool)/g**	(7.4±0.7)^bB^	(11.3±0.9)^aA^	(2.5±0.2)^cB^	(6.8±1.3)^bA^
***w*(lipid in stool)/%**	(0.82±0.04)^dB^	(1.32±0.06)^cA^	(4.08±0.07)^aA^	(2.1±0.1)^bB^
***w*(heart)/(g/100 g)**	0.26±0.01	0.25±0.01	0.28±0.01	0.27±0.01
***w*(liver)/(g/100 g)**	2.52±0.08	2.41±0.08	2.48±0.05	2.41±0.03
***w*(epidid. fat)/(g/100 g)**	(0.71±0.08)^b^	(0.70±0.05)^b^	(1.07±0.07)^aA^	(0.75±0.04)^bB^
***w*(retrop. fat)/(g/100 g)**	(0.57±0.06)^bc^	(0.46±0.04)^c^	(0.9±0.1)^a^	(0.70±0.04)^ab^

C=group with a standard diet (control), CB=group with standard diet supplemented with 10 % bract flour, CAF=group with cafeteria diet, CAFB=group with cafeteria diet supplemented with 10 % bract flour, NAL=naso-anal length, epidid. fat=epididymal fat, retrop. fat=retropenitoneal fat. Values were presented as mean±standard error of the mean (SEM), *N*=8. Different lowercase letters in the same row indicate significant differences at p<0.05 among groups (ANOVA and Tukey’s test). Different capital letters in the same row indicate significant differences at p<0.05 between groups (C+CB) and (CAF+CAFB) (*t*-test). Dietary intake, water and stool mass were calculated per animal per day. Tissue mass was related to final body mass. *Feed consumption was checked per box containing 2–3 animals, after which the average consumption per animal was taken. **Data are presented as mean±standard deviation of the mean (S.D.)

Pastore *et al*. (*35*) also observed no statistical difference in mass gain of male Wistar rats fed for 12 weeks with a cafeteria diet containing corn crackers, toasted peanuts and chocolate compared to the control group. As shown in Table 2, the CB group consumed the highest amount of feed in grams ((23.90±0.02) g/day). Although the CAF diet was theoretically more palatable, the CAF group consumed a smaller amount ((14.2±0.9) g/day). The reduced hypercaloric or high-fat diet intake compared to the control group has already been previously demonstrated (*36*). The caloric density of the CAF diet and satiety due to the caloric content and monotony may have decreased CAF diet intake (*37*).

The CAFB group showed higher feed intake and energy value consumed per day ((20.2±0.6) g/day and (319.65±25.10) kJ/day, respectively) than the CAF group ((14.2±0.9) g/day and (238.06±20.92) kJ/day, respectively), even though the final mass did not change significantly (Table 2). Between control and CB groups, there was no statistically significant difference in diet consumption; however, the CB group consumed lower energy density ((270.70±12.55) kJ/day) (Table 2). The groups with bract flour added to the diet (CB and CAFB) showed the highest water intake and stool mass, which may be due to a large amount of dietary fibre in bract flour, with a greater emphasis on insoluble fibre. This fibre is characterised by retaining water in the intestine with a consequent increase in the faecal bolus volume (*4*). Liyanage *et al*. (*19*) also found a significant increase in the faecal mass of banana-inflorescence-fed rats.

Comparing the faecal lipid excretion amounts between the control and CB groups, it was observed that adding bract flour to the standard diet resulted in a higher percentage of lipid elimination. Dietary fibre and phenolic compounds reduce intestinal absorption of lipids due to greater faecal fat excretion. Soluble fibre increases viscosity, which can slow down the absorption of some nutrients. In contrast, insoluble fibre can increase intestinal motility by reducing the contact of nutrients with cells that perform absorption (*4*, *38*).

No statistical difference among samples was found in heart and liver relative mass (Table 2). The CAF group showed the highest epididymal fat value ((1.07±0.07) g/100 g), differing from the other groups at the statistical level. The CB group showed the lowest retroperitoneal fat value ((0.46±0.04) g/100 g). The results corroborate the findings of Pastore *et al*. (*35*), who reported increased visceral fat in animals fed a high-calorie diet, without significant differences in the final mass compared to the control group.

The biochemical and haematological analyses are shown in Table 3. The fasting glucose values are within the results range observed by Giknis and Clifford (*39*). For fasting glucose, CAF ((113.6±4.2) mg/100 mL) and CB ((90.3±3.6) mg/100 mL) diets had the highest and lowest values, respectively, and were statically significant. The result was possibly due to the higher fibre mass fraction in the CB diet than in the CAF diet. Liyanage *et al.* (*19*) also observed reduced concentration of blood glucose in rats supplemented with banana inflorescence. Finally, Vilhena *et al*. (*12*) verified the antidiabetic activity of extracts and fractions from the bracts and flowers of *Musa* × *paradisiaca* in treated diabetic rats compared to the untreated diabetic group.

**Table 3:** Biochemical and haematological parameters for different experimental groups

**Parameter**	**C**	**CB**	**CAF**	**CAFB**
***γ*(GLU)/(mg/100 mL)**	(99.8±4.6)^ab^	(90.3±3.6)^b^	(113.6±4.2)^a^	(100.8±3.4)^ab^
***γ*(CHO)/(mg/100 mL)**	(42.0±1.8)^bc^	(34.8±2.6)^c^	(57.5±2.3)^aA^	(47.0±3.2)^bB^
***γ*(TG)/(mg/100 mL)**	(25.9±2.4)	(27.1±1.8)	(29.3±1.8)	(31.4±2.4)
***N*(AST/(U/L)**	(97.9±7.2)^a^	(80.6±4.0)^ab^	(96.7±6.7)^a^	(71.4±4.0)^b^
***N*(ALT)/(U/L)**	(316±1.8)^a^	(27.7±1.7)^ab^	(31.8±0.9)^aA^	(24.0±0.9)^bB^
***γ*(ALB)/(g/L)**	(38.7±1.5)^ab^	(35.0±2.0)^b^	(44.2±0.7)^aA^	(37.2±1.6)^abB^
***N*(AMYL)/(U/L)**	(1970±115)^aA^	(1514±116)^bB^	(2218±98)^a^	(1991±76)^a^
***γ*(UA)/(mg/100 mL)**	(0.75±0.05)^b^	(0.70±0.04)^b^	(1.03±0.07)^aA^	(0.66±0.04)^bB^
***γ*(CRE)/(mg/100 mL)**	0.32±0.04	0.36±0.03	0.35±0.04	0.33±0.03
***γ*(TP)/(g/L)**	56.3±2.4	61.5±3.6	58.3±2.2	59.2±2.1
***N*(leukocyte)/(·10^3^/μL)**	4.4±0.5	3.2±0.5	4.220.6	4.6±0.6
***w*(haematocrit)/%**	42.5±1.7	41.5±1.4	39.7±1.3	40.0±2.0
***N*(platelet)/(·10^3^/μL)**	696±32	614±40	596±41	698±284
***w*(lymphocyte)/%**	81.3±1.0	78.7±1.6	77.4±1.8	82.1±1.6

C=group with a standard diet (control), CB=group with standard diet supplemented with 10 % bract flour, CAF=group with cafeteria diet, CAFB=group with cafeteria diet supplemented with 10 % bract flour, GLU=glucose, CHO=total cholesterol, TG=triglycerides, AST=aspartate aminotransferase, ALT=alanine aminotransferase, ALB=albumin, AMYL=amylase, UA=uric acid, CRE=creatinine, TP=total proteins. Values were presented as mean value±standard error of the mean (SEM), *N*=8. Different lowercase letters in the same row indicate significant differences at p<0.05 among groups (ANOVA and Tukey’s test). Different capital letters in the same row indicate significant differences at p<0.05 between groups (C+CB) and (CAF+CAFB) (*t*-test)

For total cholesterol analysis, significant variations were observed. The CB group showed a low total cholesterol value ((34.8±2.6) mg/100 mL), statistically different from CAF and CAFB and lower than the normal range for Wistar strain rats obtained by Giknis and Clifford (*39*). The CAFB group ((47.0±3.2) mg/100 mL) showed a statistically lower value than the CAF group ((57.5±2.3) mg/100 mL). This reduction may be due to the presence of bract flour dietary fibre in the CAFB diet. The reduction of plasma cholesterol concentrations may be due to the binding ability of the insoluble fibre to bile acids and bile acid intestinal absorption reduction by soluble fibre, thereby promoting hepatic cholesterol depletion as a substrate for the new bile acid synthesis lost in the stool (*40*).

In a study that evaluated the hypolipidemic effect of jaboticaba (*Myrciaria cauliflora*) peel flour in rats fed a moderately high-fat diet, the authors concluded that these effects could be attributed, at least in part, to its fibre and phenolic composition of the flour (*15*).

The CAFB diet contributed to decreased AST ((71.4±4.0) U/L) and ALT ((24.0±0.9) U/L) activities when compared to the CAF ((96.7±6.7) and (31.8±0.9) U/L) and control ((97.9±7.2) and (31.6±1.8) U/L) groups, which were similar to the CB group ((80.6±4.0) and (27.7±1.7U/L)) (Table 3). In addition, a study that evaluated a high-fat and high-fructose diet with the addition of chia seed (*Salvia hispanica* L.) found a reduction in liver damage markers AST and ALT compared to the group that received only a high-fat and high-fructose diet (*36*).

When analysing rats fed with high cholesterol diet with and without banana inflorescence supplementation, Liyanage *et al.* (*19*) found lower serum AST and total cholesterol values in the group with the supplementation. The authors showed that banana inflorescence had hepatoprotective ability by reducing oxidative stress.

According to Maya-Cortés *et al.* (*41*), serum albumin level variation may indicate liver disease. The group that received the CAF diet had the highest statistical value of serum albumin levels ((44.2±0.7) g/L) when compared to the CAFB ((37.2±1.6) g/L) and CB groups ((35.0±2.0) g/L) (Table 3). These results may explain a possible hepatoprotective effect of banana bract flour when comparing the CAF and CAFB groups. The CB group showed a statically lower value for serum amylase enzyme than the other groups ((1514±117) U/L). This result may be justified by the presence of insoluble fibre in the diet, reducing digestive enzyme activity (*4*). The amylase enzyme analysis results showed that only the CAF group had a value slightly above normal, according to Giknis and Clifford (*39*).

The CAF group showed uric acid concentrations statistically higher than the other groups ((1.03±0.07) mg/100 mL). Fructose is added to industrialised product formulations, such as soft drinks and biscuits. Excessive fructose consumption may be related to increased uric acid (*42*). The CAF diet contained food rich in fructose, such as wafer biscuits, and access to soft drink *ad libitum*. Thus, these foods may have contributed to the concentration increase of uric acid in the CAF group, and the banana bract flour may have had a protective effect in the CAFB group.

No significant variations were found between control and experimental groups in creatinine, total protein, and haematological analyses (p>0.05). According to Giknis and Clifford (*39*), the values found in this study are within the normal range for healthy rats at this age.

**Fig. 2:**
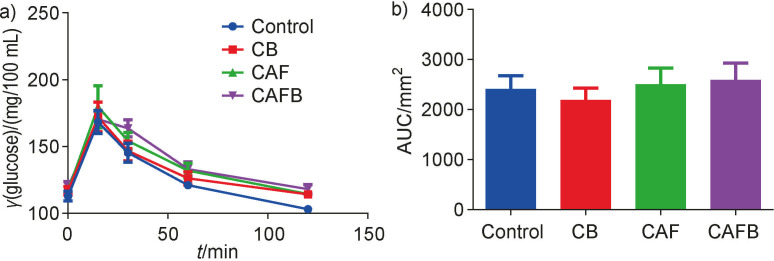
Intraperitoneal glucose tolerance test (ipGTT). Values were presented as mean±standard error of the mean (SEM), *N*=8: a) mean blood glucose concentrations at time zero (basal), 15, 30, 60 and 120 min after intraperitoneal infusion of glucose solution, and b) glucose area under the curve (AUC) during ipGTT). C=standard diet group, CB=standard diet group supplemented with 10 % bract flour, CAF=high-calorie diet group, CAFB=high-calorie diet group supplemented 10 % bract flour

When analysing the glucose tolerance test (Fig. 2a), no statistical difference was found between the control and experimental groups in the area under the curve (AUC) (Fig. 2b). Such a result demonstrates that bract flour supplementation and cafeteria type high-calorie diet did not modify the glucose tolerance of the tested animals.

**Fig. 3:**
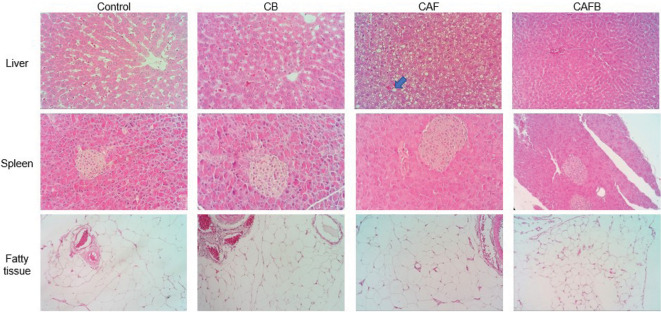
Tissue photomicrographs of control, CB, CAF and CAFB groups stained with hematoxylin and eosin (HE) (20×). The blue arrow on the CAF group liver indicates a large cytoplasmic vacuole (macrovesicular steatosis) in the hepatocyte, HE 40×. Control=standard diet group, CB=standard diet group supplemented with 10 % bract flour, CAF=cafeteria (high-calorie) diet group, CAFB=cafeteria (high-calorie) diet group supplemented with 10 % bract flour

Although there is no difference in organ mass between groups, the CAF group had higher values for AST, ALT and albumin. In this group, the histological evaluation of the liver (Fig. 3) revealed hepatocytes with small cytoplasmic vacuoles and the nucleus in the central position (microvesicular steatosis) or hepatocytes showing a large cytoplasmic vacuole and the nucleus shifted to the cell periphery (macrovesicular steatosis). These changes were either random, centrilobular, or in multifocal areas of the analysed microscope slides. Silva *et al*. (*43*) found that a high-fat diet increased hepatic fat deposition in animals, causing clinical manifestations such as changes in organ function and steatosis. For the remaining groups, no hepatic changes were observed. Thus, the bract flower may have exerted a hepatoprotective effect on the CAFB group. In addition, banana bract flour has many phenolic compounds that inhibit lipid peroxidation and tissue oxidation, especially in the liver, due to its antioxidant properties, thus exerting a hepatoprotective effect (*44*).

The study of Loubet Filho *et al*. (*45*) evaluated the effects of the guavira (*Campomanesia* sp.) fruit industrial residue in rats fed with a high-calorie diet. The consumption of guavira industrial residue flour attenuated non-alcoholic fatty liver disease caused by a high-calorie diet. The authors suggested that this effect may be due to this flour high fibre content and bioactive compounds (*45*). Interestingly, in their study, there was no statistical difference in the oral glucose tolerance test between groups.

Another study investigated the effects of 4 % jaboticaba (*Myrciaria cauliflora*) peel powder supplementation on cholesterol metabolism and hepatic steatosis of rats with high-fat diets (*46*). In agreement with what was observed in this study, the authors of the study with jaboticaba observed an increase in lipid excretion in the faeces, improvement in the serum lipid profile, reduction in serum total cholesterol concentration, reduction in serum AST activity and attenuation of hepatic steatosis. These effects were suggested to be related to the presence of fibre and phenolic compounds in the powder supplementation (*46*).

Histological analysis of the pancreas and adipose tissues revealed no changes between groups after 12 weeks of intervention (Fig. 3). These results corroborate a study that evaluated male Wistar rats fed with high fat and sugar diets for 6, 12 and 24 weeks and showed an increase in adipocyte expression only for the group that received the diet for 24 weeks (*47*).

**Fig. 4:**
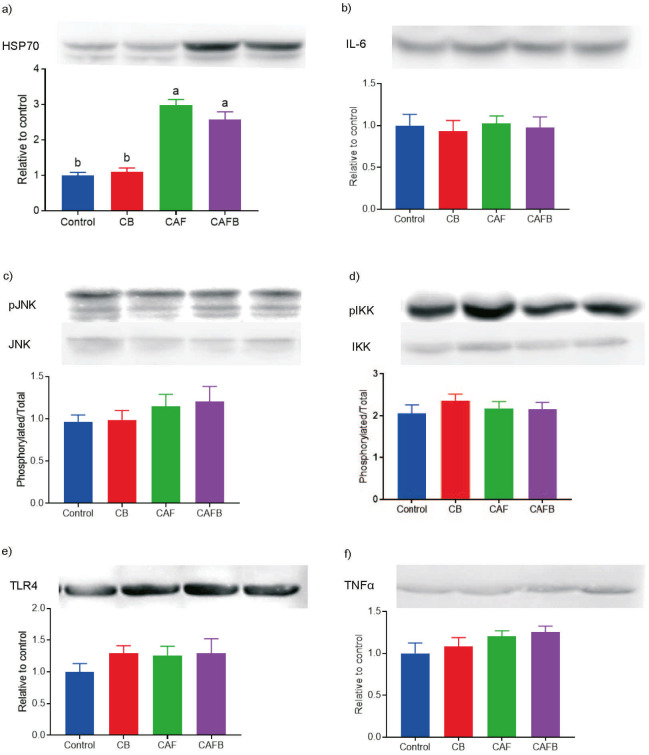
Inflammatory protein targets in the muscle of Wistar rats after a 12-week experimental design: a) HSP70=heat shock protein 70, b) IL-6=interleukin 6, c) JNK=C-Jun NH2-terminal kinase, d) IKK=kB kinase, e) TLR4=toll-like receptor 4, and f) TNFα=tumour necrosis factor alpha. C=group with a standard diet (control), CB=group with standard diet supplemented with 10 % bract flour, CAF=group with cafeteria (high-calorie) diet, CAFB=group with cafeteria (high-calorie) diet supplemented with 10 % bract flour

Western blot analyses showed that the supplementation with banana bract flour did not change HSP70 expression (Fig. 4). However, a significant difference was seen between the control and high-calorie diet group. Similar results were found in Wistar rats that received a high-fat, high-fructose diet for 12 weeks, with HSP70 expression increased in the soleus muscle compared to the group control (*36*). In the same study, no statistical differences were seen in the expression of HSP70 between the group that received a high-fat, high-fructose diet *vs* a high-fat, high-fructose diet supplemented with chia seeds for 12 weeks. Stygar *et al*. (*48*) suggested that a stressor factor, such as a high-calorie diet, may induce known responses in the antioxidant defence system and increase HSP70 concentrations, which may be an adaptive response leading to compensation. Thus, increased HSP70 expression in the muscle could prevent disturbances in metabolic homeostasis by reducing inflammation and improving glucose tolerance, mitigating the damage caused by a high-fat diet (*49*).

Increased HSP expression may inhibit the inflammatory kinase phosphorylation and activation, such as c-Jun NH2-terminal kinase (JNK) and kB kinase-B (IKKβ), which are usually high in obesity and showed impaired insulin signalling pathways. For this reason, an HSP70 increase could protect the individual from insulin resistance and remedy diet-induced glucose intolerance (*50*). However, as previously mentioned in this study, no statistically significant difference was found in the glucose tolerance test. Moreover, no statistical differences were found when evaluating the ratios of the phosphorylated and total JNK and IKK protein forms. In the study of Henstridge *et al.* (*50*), mice fed with a high-calorie diet for 10 weeks showed overexpression of HSP72, and no JNK activation in the skeletal muscle, despite increased mass and body fat. They concluded that HSP overexpression was related to a reduction in the inflammatory markers in the high-calorie diet group and also prevented insulin resistance in this group.

According to Stygar *et al*. (*48*), reports on HSP levels in different pathological situations and stimulation are often confusing and contradictory. The authors suggest that the system response measured by HSPs depends on the intensity and duration of a stimulation and the overall characteristics of a stressor. These findings align with the results found in our study, and the increased HSP70 expression may be linked to the inhibition of the inflammatory markers JNK, IKKβ, TLR4, IL6 and TNFα in the groups fed with a high-calorie diet. The time of the evaluation of these animals could have coincided with the compensatory phase observed in the literature, in which an increase in HSSP70 as a cellular defence strategy occurs as an attempt to minimise possible damages caused by a high-calorie diet. Interventions leading to increase in the HSP could be key to preventing inflammatory and metabolic damage caused by obesity. More studies are needed for a thorough understanding and interpretation of this phenomenon.

## CONCLUSIONS

The analysis of banana (*Musa* ssp.) bract flour showed its considerable antioxidant value with high phenolic content. In addition, the *in vivo* study suggested that bract flour may be useful as a preventive agent for pathological damage associated with a high-calorie diet. It was noted that the animals supplemented with the flour had lower body fat, glucose and total cholesterol values in serum and higher values of lipid volume and faecal excretion. Furthermore, a bract flour hepatoprotective effect was observed. However, further studies should be conducted to analyse its nutritional impact on the human diet and the percentage of supplementation, as well as to evaluate the action of HSP70 as a strategy to prevent the damage caused by obesity.
